# Structural and Biological Features of G-Quadruplex Aptamers as Promising Inhibitors of the STAT3 Signaling Pathway

**DOI:** 10.3390/ijms24119524

**Published:** 2023-05-30

**Authors:** Veronica Esposito, Daniela Benigno, Ivana Bello, Elisabetta Panza, Mariarosaria Bucci, Antonella Virgilio, Aldo Galeone

**Affiliations:** Department of Pharmacy, University of Naples Federico II, Via D. Montesano 49, I-80131 Naples, Italy; verespos@unina.it (V.E.); daniela.benigno@unina.it (D.B.); ivana.bello@unina.it (I.B.); e.panza@unina.it (E.P.); mrbucci@unina.it (M.B.); galeone@unina.it (A.G.)

**Keywords:** G-quadruplex, aptamers, antiproliferation, STAT3

## Abstract

In this paper, we investigate the structural and biological features of G-quadruplex (G4) aptamers as promising antiproliferative compounds affecting the STAT3 signalling pathway. Targeting the STAT3 protein through high-affinity ligands to reduce its levels or activity in cancer has noteworthy therapeutic potential. T40214 (**STAT**) [(G_3_C)_4_] is a G4 aptamer that can influence STAT3 biological outcomes in an efficient manner in several cancer cells. To explore the effects of an extra cytidine in second position and/or of single site-specific replacements of loop residues in generating aptamers that can affect the STAT3 biochemical pathway, a series of **STAT** and **STATB** [GCG_2_(CG_3_)_3_C] analogues containing a thymidine residue instead of cytidines was prepared. NMR, CD, UV, and PAGE data suggested that all derivatives adopt dimeric G4 structures like that of unmodified T40214 endowed with higher thermal stability, keeping the resistance in biological environments substantially unchanged, as shown by the nuclease stability assay. The antiproliferative activity of these ODNs was tested on both human prostate (DU145) and breast (MDA-MB-231) cancer cells. All derivatives showed similar antiproliferative activities on both cell lines, revealing a marked inhibition of proliferation, particularly at 72 h at 30 µM. Transcriptomic analysis aimed to evaluate **STAT**’s and **STATB**’s influence on the expression of many genes in MDA-MB-231 cells, suggested their potential involvement in STAT3 pathway modulation, and thus their interference in different biological processes. These data provide new tools to affect an interesting biochemical pathway and to develop novel anticancer and anti-inflammatory drugs.

## 1. Introduction

G-quadruplexes (G4s) are among the most stable nucleic acid structures and are characterized by a large conformational diversity [1]. Their existence in vivo and their functional roles are proved [2] and they are formed by interactions of repetitive G-rich tracts in genomic DNA and RNA sequences [3]. The folded G4 sequence consists of stacked G-tetrads, creating a central core of four strand segments connected by single-stranded loops. A G-tetrad is a planar arrangement of four guanine residues from different G-tracts linked by Hoogsteen hydrogen bonds. In addition to the endogenous G4s that are almost recognized as integral structures within the non-coding genome and transcriptome [4], which play a key role in numerous biological processes mainly through their interaction with several cellular proteins [5,6], very promising exogenous G4s are known, due to their ability to modulate the activity of different protein target. Indeed, a significant number of aptamers selected by the combinatorial technique Systematic Evolution of Ligands by EXponential enrichment (SELEX) towards a variety of protein targets [7] adopt G4 structures (G4 aptamers). The remarkable thermal stability of G4s provides them with a moderate resistance to nucleases, which prolongs the permanence of these aptamers in the bloodstream. Due to their advantageous properties, G4 aptamers can be considered an interesting category of molecules with several potential applications in therapeutics, diagnostics, food quality control, environmental monitoring, and drug development and delivery [8].

Among the G4s endowed with notable biological properties, G4 aptamers can interact with some cellular proteins involved in oncogenesis and cancer progression, and can be considered particularly promising pharmacological agents. A remarkable example is AS1411, a nucleolin-targeting aptamer that reached Phase 2b clinical trials for acute myeloid leukaemia and renal cell carcinoma; however, despite a good tolerance and safety profile, its trial was terminated due to its suboptimal pharmacokinetics (rapid clearance) and low potency [9,10]. Further interesting aptamers belonging to this category are T40214 and T40231 (sequences (G_3_C)_4_ and G_2_T(G_3_T)_2_G_3_, respectively), targeting Signal Transducer and Activator of Transcription 3 (STAT3) [11]. This protein is a key mediator of the oncogenic signaling that is frequently activated in many types of human cancer, where it contributes to tumor cell growth and resistance to apoptosis through the upregulation of genes encoding apoptosis inhibitors (Bcl-xL, Mcl-1, and survivin), cell cycle regulators (cyclin D1 and c-myc), and inducers of angiogenesis (VEGF) [12]. Indeed, increasing evidence has shown that STAT3 is constitutively activated in 82% of prostate cancers, 70% of breast cancers, over 90% of head and neck cancers, and more than 50% of lung cancers [13,14,15,16]. In addition, further recent studies have highlighted the prognostic significance of STAT3 levels in invasive breast cancer [17] and chordomas [18]. Therefore, STAT3 can be considered a promising target for anticancer drug discovery.

Similarly to other STAT proteins, STAT3 is localized within the cytoplasm of resting cells and activated in response to the binding of several ligands, including cytokines and other factors, to their cognate cell surface receptors (e.g., IL-6R). STAT3 activation generates dimers, which translocate to the nucleus, where they bind to DNA-response elements in the promoters of target genes and activate specific gene-expression programs [19]. STAT3 activation contributes to the expression of antiapoptosis proteins, such as Bcl-xL and Mcl-1, thus decreasing natural apoptotic cell death and favouring cancer cell growth [20,21].

**Figure 1:**
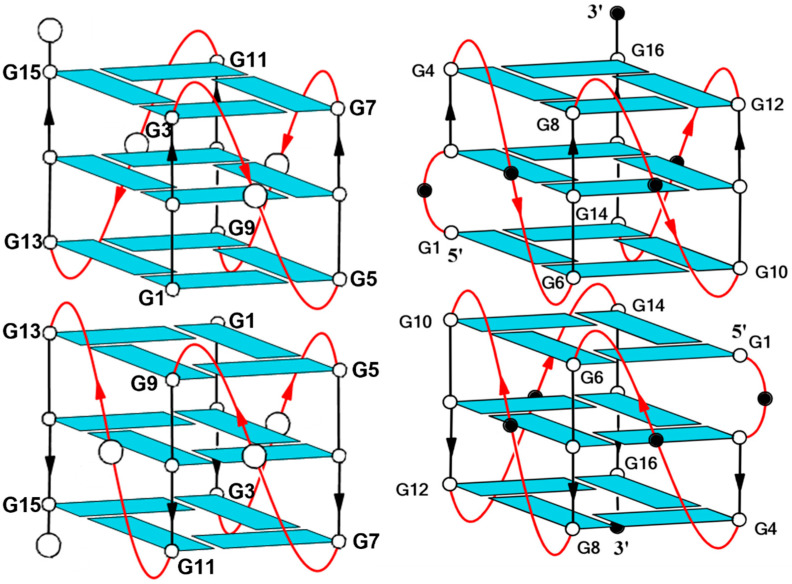
Schematic structures of the parallel-stranded dimeric G-quadruplexes formed by T30923/**STAT** (on the **left**) and T30175/**STATB** (on the **right**). Black or white circle in the loop represent thymidines (in T30923 and T30175) or cytidines (in **STAT** and **STATB**).

Therefore, targeting the STAT3 protein through high-affinity ligands with the aim of reducing its levels or activity in cancers has noteworthy therapeutic potential. T40214 (**STAT**) is a G4 aptamer [(G_3_C)_4_] that can influence STAT3 biological outcomes in an efficient manner in several cancer cell lines and tumor xenografts [11,22], forming a G4 conformation characterized by parallel strands, three G-tetrads and three propeller-shaped loops [23]. Another G4 aptamer, namely T40231 [G_2_T(G_3_T)_2_G_3_], directly targets STAT3 protein and dramatically reduces tumor cell growth by markedly enhancing the apoptosis of prostate and breast cancer cells in which STAT3 is constitutively activated [24]. Interestingly, a recent study [25] showed that a further G4-forming aptamer characterized by almost the same sequence as T40231, namely T30923 [(G_3_T)_4_] (INT), is able to efficiently link the interleukine cell surface receptors (IL-6R) which, in turn, activate STAT3. Therefore, all these G4-forming aptamers are implicated in the same bio-molecular pathway. Furthermore, it should be noted that T30923 adopts a parallel G4 structure strictly resembling that of **STAT** [23] (Figure 1). Concerning G4 anticancer aptamers, in a recent paper [26], we have reported, for the first time, the antiproliferative properties of another G4-forming aptamer, namely T30175 [GTG_2_(TG_3_)_3_T] (INTB), whose sequence and structure are closely correlated with those of T30923 [23,27,28]. In fact, their sequences differ only in a T residue, and both adopt a dimeric 5′-5′ end-stacked G4 structure, in which each parallel G4 monomer of the complex contains three G-tetrads and three single-thymidine reversed-chain loops; however, T30175 is characterized by an additional bulge loop formed by the extra thymidine in the second position of the sequence (Figure 1).

Most G4 aptamers are characterized by peculiar structural features; their stability is principally due to a compact core, formed by stacked G-tetrads, while the loop residues are mostly involved in the interaction with the target protein. Consequently, when designing modified aptamers to modulate their biological properties without affecting their stability, in most cases, modifications are focused on the loop residues directly involved in the aptamer–target interaction. Therefore, to explore the effects of single site-specific replacements of the loop residues in generating aptamers that can affect the STAT3 biochemical pathway, a series of **STAT** analogues containing a thymidine (T) residue instead of the single cytidines (C) of the sequence was prepared (Table 1). Furthermore, a second series of derivatives containing the same single-site modification was synthesized, starting from a new parent aptamer, namely **STATB** [GCG_2_(CG_3_)_3_C] (Table 1). This oligonucleotide sequence differs from that of **STAT** only by an extra cytidine in second position, similarly to T30175 versus T30923, and was designed considering the recently identified antiproliferative activity of T30175, whose structure is closely related to that of T30923, which shares the same bio-molecular pathway as **STAT**. All derivatives reported in Table 1 were investigated in terms of their structural and biological properties, with the aim of identifying new aptamers endowed with the ability to affect the biochemical pathway in which different target proteins, namely IL-6R and STAT3, are involved.

## 2. Results

### 2.1. NMR Spectroscopy

A critical issue in the present study is the evaluation of the ability of the ODN variants to fold in parallel dimeric G-quadruplex structures resembling that of the reference sequence [23]. These measurements are essential to prevent any misinterpretation of the biological activity results attributable to the presence of conformations different from that of the parent one.

**Figure 2:**
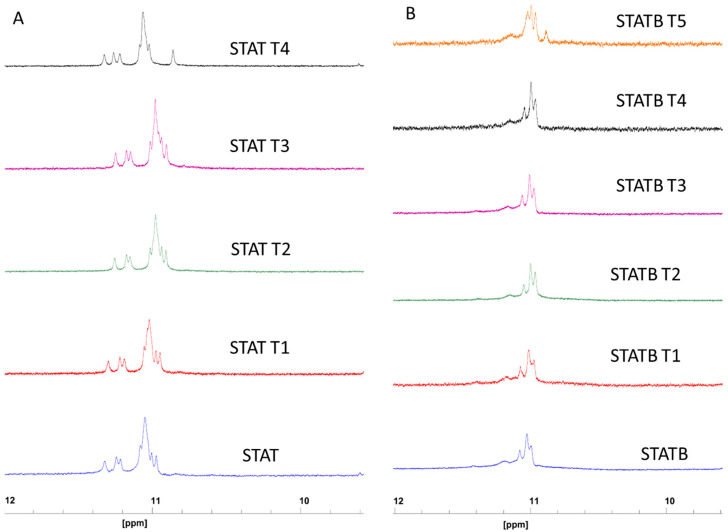
Imino proton regions of the 1H-NMR spectra (700 MHz) of **STAT** (**A**) and **STATB** (**B**) series. See Section 4 for experimental details.

To address this point, all the modified ODNs containing a single T that replaces a C loop residue, one at a time, were individually analysed by 1H-NMR and compared with their respective counterparts, characterized by all-cytidine loops (**STAT** and **STATB**) (Figure 2). A straightforward comparison of the imino proton regions diagnostic of the presence of G-quadruplex structures (10.5–12.0 ppm) strongly suggests that the G-quadruplex structures adopted by all derivatives are very similar to those of their respective reference sequences, showing related signal distribution independently of the T loop residue position in the sequence and despite the extensive overlapping.

### 2.2. CD Spectroscopy

**Figure 3:**
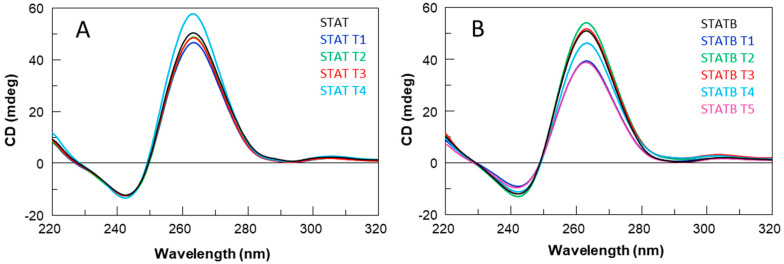
CD spectra at 20 °C of **STAT** (**A**) and **STATB** (**B**) series. See Section 4 for experimental details.

To support 1H-NMR data, the CD spectra of all derivatives were registered and compared with those of their respective reference sequences (**STAT** and **STAB**) (Figure 3A,B). Regardless of slight variations in intensity, all profiles are almost superimposable on each other, showing a minor negative band at 242 nm and a major positive band at 263 nm, typical features of a parallel folding pattern in which all guanosines adopt an *anti*-glycosidic conformation. CD data strongly suggest that all the examined ODNs adopt G4 structures strictly resembling that of the parent aptamer T40214, in agreement with NMR results.

Moreover, the thermal stability of all G4 derivatives was determined by CD melting experiments, acquiring CD heating profiles in the NMR buffer conditions (Figure S1A,B) that were the same as those used in biological assays. Regarding the G4 structures of **STATB** series, the registered melting temperatures above 80 °C, as in the case of the original structure, suggested no appreciable influence of thymine on the G4 thermal stability. On the other hand, the unchanged CD signal up to about 80 °C and the absence of a sigmoidal melting profile for all the sequences of **STAT** series suggest that the high structural stability of the parent G4 structure was mostly preserved.

Nevertheless, some differences in the structural stability of **STAT** analogues could be noted by recording the CD melting temperatures (T_m_) of all the samples (**STAT** and **STATB**) at very low potassium ion concentrations (5 mM KCl) (Table 1, Figure S2A,B).

The CD data acquired in these conditions denoted a remarkable stability for all structures, which was higher than that of the respective parent G4 complex in almost all cases.

All the data indicated that, under the biological assay conditions (37 °C), all G4 structures were retained, adopting very stable conformations.

### 2.3. UV Thermal Difference Spectra (TDS)

**Figure 4:**
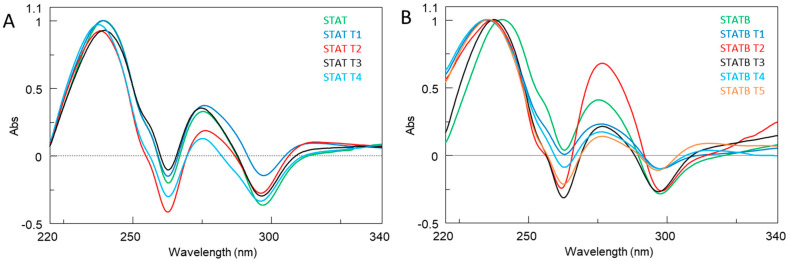
Normalized TDS profiles of **STAT** (**A**) and **STATB** (**B**) series. See Section 4 for experimental details.

The formation of quadruplexes was further confirmed by UV thermal difference spectra (TDS). Since this technique reflects the subtleties of base stacking interactions, it is often used to compare G-quadruplex structures. Figure 4 shows the TDS profiles of the analysed quadruplex structures and their reference counterparts. The shape of the TDS profiles was relatively similar and very close to those previously reported for these complexes, sharing a positive band around 275 nm and a negative one around 295 nm. These features are common for most of the G-quadruplex structures investigated by this technique. On the other hand, there were slight differences in the region between 220 and 270 nm, as observed in the comparison of other types of G quadruplexes [29]. Thus, the TDS results are in agreement with the CD data, indicating the presence of G-quadruplex structures.

### 2.4. Polyacrylamide Gel Electrophoresis (PAGE)

It should be noted that the previous experiments, although informative, do not provide clear evidence of the presence of dimers for the analysed sequences. In order to address this aim, we examined them using Polyacrylamide Gel Electrophoresis (PAGE) (Figure S3), using INT and INTB, which have already been proven to fold into 5′-5′ dimers of two stacked parallel G-quadruplexes [27,28], as band motility dimeric references for the **STAT** and **STATB** series, respectively. Instead, TT-INT and TT-INTB, in which the dimer formation is avoided by the extra thymidines in 5′, were used as monomeric references [28]. The PAGE results unambiguously revealed that all G4 samples show slower-migrating bands typical of dimeric structures, while TT-INT and TT-INTB show faster migrating bands, since they mainly fold into monomeric forms. The PAGE results are in agreement with the data obtained by the other techniques, all pointing to the presence of G4 structures for the aptamers that are strictly superimposable on that assumed by the reference ODNs.

### 2.5. Nuclease Stability Assay

**Figure 5:**
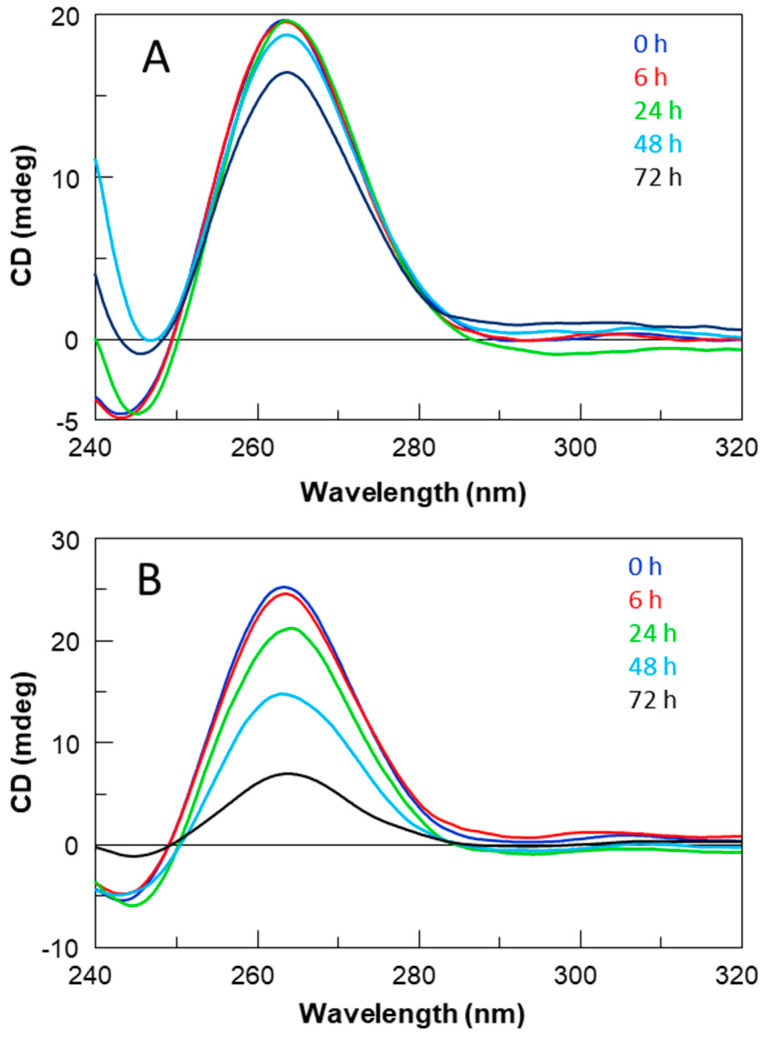
CD spectra of **STAT** (**A**) and **STATB** (**B**) in 10% Fetal Bovine Serum (FBS) diluted with Dulbecco’s Modified Eagle’s Medium (DMEM), registered at a different time at 37 °C. See the main text and Section 4 for details.

Since the aptamer vulnerability due to nucleases digestion is the major element restricting their therapeutical application, to test the resistance in biological environments, all the investigated ODNs underwent to a degradation assay in fetal bovine serum (FBS) and were analyzed by circular dichroism [30] (Figure 5, Figures S4 and S5). To prove the persistence of the CD signal attributable to undegraded G4 folded species over time, CD spectra of all ODNs were registered in 240–320 nm region at 0, 6, 24, 48 and 72 h at 37 °C in 10% FBS, subtracting the background scan (10% FBS in DMEM). The CD spectra of each sample at different times of incubation preserved the typical CD profile of parallel G4 in which all guanosines adopt *anti*-glycosidic conformations. Although a time-dependent reduction in band intensities indicates degradation to some extent, a notable stability to serum nucleases of **STAT** series derivatives was plainly observable. CD spectra at 72 h, under the experimental conditions, proved the persistence of about 80–85% of folded structures (Table S1). Otherwise **STATB** series quadruplexes, under the same conditions, showed a lower nuclease resistance, since approximately 28–44% of G4 structured species were still present at 72 h (Table S2).

### 2.6. Antiproliferative Activity

**Figure 6:**
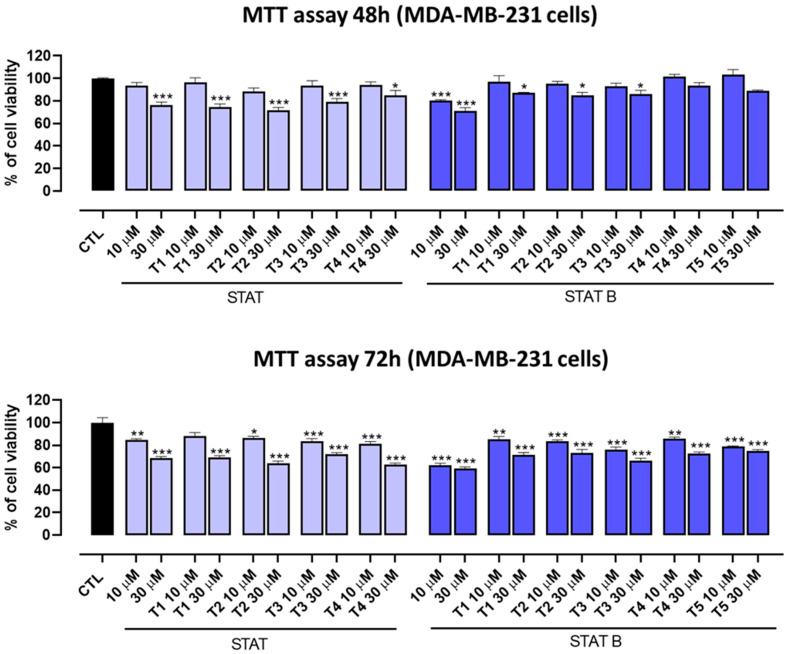
Effect of **STAT** and **STATB** series on MDA-MB-231 cell proliferation. Cell proliferation was measured using the MTT assay and evaluated at 48 and 72 h. Each experiment (*n* = 3) was run in quadruplicate. * *p* < 0.05; ** *p* < 0.01; *** *p* < 0.001 vs. CTL.

The potential anticancer activity of **STAT** and **STATB** series quadruplexes was examined regarding the proliferative capacity of MDA-MB-231 cells, a validated in vitro model of human breast cancer, using MTT assay. MDA-MB-231 cells were exposed to **STAT** and its four distinct derivatives, and to **STATB** and its five analogues (Table 1). Each compound was tested at 10 and 30 µM for 48 and 72 h. Cells not exposed to the investigated derivatives were used as control. Our results revealed that both **STAT** and **STATB** series quadruplexes at 30 µM and after 48 h exerted antiproliferative effects in MDA-MB-231 cells, while only **STATB** at 10 µM significantly inhibited the proliferation of cells. After 72 h, a significant inhibition of cell proliferation (inhibition percentages ranging from about 10 to 40%) was observed with all the **STAT** and **STATB** series quadruplexes used at both 10 and 30 µM (Figure 6). Particularly, the most marked inhibition effect (40%) was produced by **STATB**, reaching a plateau between 10 µM and 30 µM and proving to be the most active even at low concentrations. Similar results were also obtained in DU145 cells, a commonly used prostate cancer cell line (Figure S6).

### 2.7. Transcriptomic Analysis of Proliferation, Inflammation, and Angiogenesis Genes

**Figure 7:**
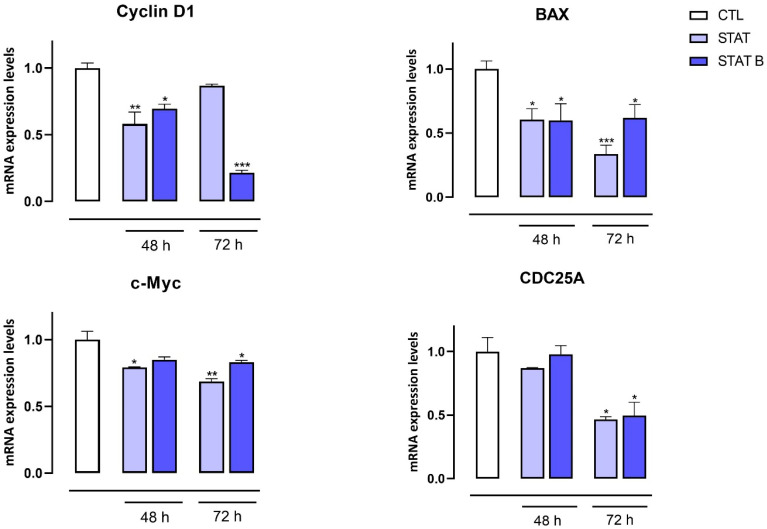
mRNA expression levels of Cyclin D1, BAX, c-Myc and CDC25A in MDA-MB-231 cells following the treatment with **STAT** and **STATB** (30 μM) for 48 and 72 h Each data point was obtained from at least three independent determinations for each experimental condition. * *p* < 0.05; ** *p* < 0.01; *** *p* < 0.001 vs. CTL.

**Figure 8:**
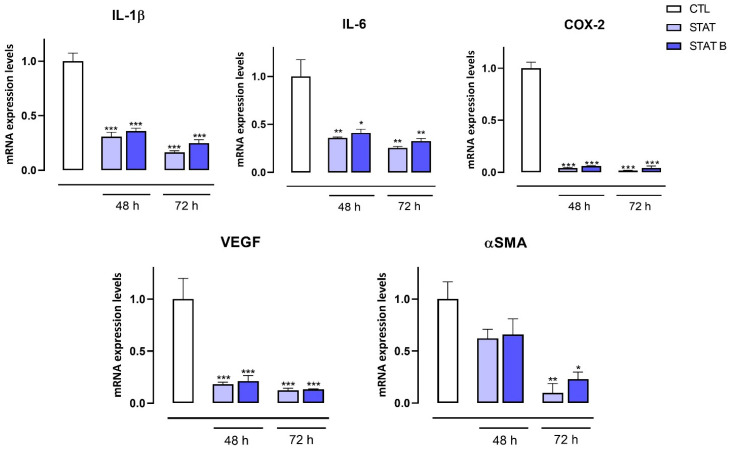
mRNA expression levels of IL-1β, IL-6, COX-2, VEGF and αSMA in MDA-MB-231 cells following the treatment with **STAT** and **STATB** (30 μM) for 48 and 72 h Each data point was obtained from at least three independent determinations for each experimental condition. * *p* < 0.05; ** *p* < 0.01; *** *p* < 0.001 vs. CTL.

STAT3 is known to be one of the most important transcriptional factors regulating the expression of key oncogenes involved in breast cancer. Recent data indicate that overexpressed and constitutively activated STAT3 is involved in the development, tumour-promoting inflammation, progression, and chemoresistance of breast cancer [31]. Therefore, to measure the expression of a large plethora of STAT3-regulated genes involved in proliferation, inflammation, and angiogenesis, we carried out a transcriptomic analysis, using quantitative PCR (qPCR), in MDA-MB-231 cells exposed to **STAT** and **STATB** at 30 µM for 48 and 72 h. In this analysis, we focused our attention on the unmodified sequence of each series, since all derivatives of the **STAT** series in an MTT assay revealed an antiproliferative activity similar to that of the parent one, while **STATB** showed better antiproliferative properties than its tested analogues. We found that the mRNA expression of cell-cycle-related genes, including Cyclin D1, BAX, c-Myc and CDC25A, was significantly reduced by both compounds (Figure 7), with a more pronounced effect of **STATB** on Cyclin D1 expression. Additionally, **STAT** and **STATB** reduced the expression of pro-inflammatory and pro-angiogenesis genes, such as IL-1β, IL-6, COX-2, VEGF and αSMA (Figure 8).

## 3. Discussion

The signal transducer and activator of transcription (STAT) proteins, particularly STAT3, are ideal targets for cancer therapy. STAT signalling is mainly regulated by interleukin-6 (IL-6) and its family members, although new pathways regulating STAT3 in cancer were recently identified [32]. The continuous activation of the IL-6/STAT3 signaling pathway has been detected in cell proliferation, survival, and invasion of many human cancers, including breast cancer [31,33]. Recent data showed that the blockage of the IL-6/STAT3 pathway results in the activation of apoptosis [34], thus suggesting that new regulators of STAT3 in tumours are important targets for potential therapeutic strategies in the treatment of cancer. Despite the evident potential of STAT3 as a target to obtain antitumour effects, to date, satisfying interventions aimed at inhibiting this protein have yet to be developed, due to the complexity of its biological functions.

Therefore, with the aim of designing new aptamers that can interfere in the biochemical pathway involving different target proteins, namely IL-6R and STAT3, we focused on the G4-aptamer T40214 (**STAT**), which was reported to bind to STAT3 with high affinity [11,22], and on its similar sequence analogue **STATB**, synthesizing two series of derivatives containing a thymidine (T) residue instead of the single cytidines (C) of the loop sequences.

All ODNs were evaluated in terms of their structural and biological properties. The collected NMR, CD, UV, and PAGE data suggest that all derivatives adopt dimeric G4 structures strictly resembling that of the unmodified aptamer T40214, endowed with a higher thermal stability than their parent, **STAT** and **STATB,** respectively, thus indicating that the single-site replacement of a C residue with a T not only preserves the original quadruplex structure but significantly contributes to its thermal stability.

Concerning the ODN biostability, which plays a key role in their applicability in the therapeutic field, **STAT** series derivatives revealed a notable resistance to serum nucleases against a lower resistance of the **STATB** series. Single-loop modifications contributed positively, since all modified ODNs showed higher percentages of undegraded species at 72 h than the unmodified sequence, particularly in the **STATB** series. These data suggest a probable correlation between biostability and thermal stability: the G-quadruplexes endowed with higher thermal stability guarantee a major amount of the folded species that are biologically more stable than the thermally less stable structures in equilibrium with a larger amount of the unstructured species being more susceptible to nucleases.

The antiproliferative activity of the investigated ODNs was tested on both human prostate (DU145) and breast (MDA-MB-231) cancer cells. Interestingly, the entire series of **STAT** and **STATB** quadruplexes reduced the proliferative capacity of the aforementioned cells, exhibiting different inhibition effects depending on time and concentration. Of note, in MDA-MB-231 cancer cells after 72 h, **STAT** and its derivatives showed the highest inhibition effects at 30 µM (inhibition percentages ranging from about 25 to 40%). After the same incubation time, the most pronounced antiproliferative activity (40%) was exhibited by **STATB**, which reached a plateau between 10 µM and 30 µM, showing the greatest efficacy at a low concentration. Next, we evaluated the effect of **STAT** and **STATB** quadruplexes on STAT3 transcriptional activity. We were able to demonstrate that both **STAT** and **STATB** compounds induce significant changes in the mRNA expression levels of genes regulating key cellular process, such as cell cycle, inflammation, and angiogenesis. In summary, this approach allowed us to demonstrate the efficacy of these novel aptamers targeting STAT3 in cancer cells.

## 4. Materials and Methods

### 4.1. Oligonucleotide Synthesis and Purification

The ODNs listed in Table 1 were synthesized by an ABI 394 DNA synthesizer using solid-phase β-cyanoethyl phosphoramidite chemistry at the 10 µmol scale. The synthesis was carried out using normal 3′-phosphoramidites (Link Technologies, Glasgow, UK). The detachment from the support and the deprotection of the oligomers were carried out by treatment with concentrated aqueous ammonia at 55 °C overnight. The combined filtrates and washings were concentrated under reduced pressure, redissolved in H_2_O, analyzed, and purified by high-performance liquid chromatography on a Nucleogel SAX column (Macherey-Nagel, Duren, Germany, 1000-8/46) using buffer A (20 mM NaH_2_PO_4_/Na_2_HPO_4_ aqueous solution (pH 7.0) containing 20% (*v*/*v*) CH_3_CN) and buffer B (1 M NaCl, 20 mM NaH_2_PO_4_/Na_2_HPO_4_ aqueous solution (pH 7.0) containing 20% (*v*/*v*) CH_3_CN); a linear gradient from 0% to 100% B for 45 min and a flow rate of 1 mL/min were used. The fractions of the oligomers were collected and successively desalted by Sep-pak cartridges (C-18). The isolated oligomers proved to be >98% pure by NMR.

### 4.2. NMR Spectroscopy

NMR samples were prepared at a concentration of approximately 1 mM in 0.6 mL (H_2_O/D_2_O 9:1 *v*/*v*) of buffer solution with 10 mM KH_2_PO_4_/K_2_HPO_4_, 70 mM KCl, and 0.2 mM EDTA (pH 7.0). All the samples were heated for 5–10 min at 90 °C and slowly cooled (10–12 h) to room temperature. The solutions were equilibrated for several hours at 4 °C. The annealing process was assumed to be complete when the ^1^H NMR spectra were superimposable on changing time. NMR spectra were recorded at 25 °C by employing a 700 MHz Bruker spectrometer (Bruker-Biospin, Billerica, MA, USA). Proton chemical shifts were referenced to the residual water signal, resonating at 4.78 ppm (25 °C, pH 7.0). Water suppression was achieved using the excitation sculpting with the gradient routine included in the “zgesgp” pulse sequence [35]. NMR data processing was done by using the vendor software TOPSPIN 4.1.4 (Bruker Biospin Gmbh, Rheinstetten, Germany).

### 4.3. CD Spectroscopy

CD samples of oligonucleotides reported in Table 1 were prepared at an ODN concentration of 50 µM using a potassium phosphate buffer (10 mM KH_2_PO_4_/K_2_HPO_4_, 70 mM KCl, pH 7.0) and submitted to the annealing procedure (heating at 90 °C and slowly cooling at room temperature). CD spectra of all quadruplexes and CD melting curves were registered on a Jasco 715 CD spectrophotometer (Jasco, Tokyo, Japan). For the CD spectra, the wavelength varied from 220 to 320 nm at 100 nm min^−1^ scan rate, and the spectra recorded with a response of 4 s, at 1.0 nm bandwidth and normalized by subtraction of the background scan with buffer. The temperature was kept constant at 20 °C with a thermoelectrically controlled cell holder (Jasco PTC-348). CD melting curves were registered as a function of temperature (range: 20 °C–95 °C) for all G-quadruplexes, annealed as previously reported, at their maximum Cotton effect wavelengths. To test the G-quadruplex thermal stabilities at low potassium concentration, samples of all ODNs were prepared at an ODN concentration of 25 µM, using a potassium phosphate buffer 1 mM KH_2_PO_4_/K_2_HPO_4_, 5 mM KCl, pH 7.0) and submitted to the annealing procedure as previously described. CD melting curves were registered as a function of temperature (range: 20 °C–95 °C) for all G-quadruplexes at their maximum Cotton effect wavelengths. The CD data were recorded in a 0.1 cm pathlength cuvette with a scan rate of 30 °C/h.

### 4.4. UV Thermal Difference Spectra (TDS)

UV samples of investigated oligonucleotides were prepared using a buffer solution: KH_2_PO_4_/K_2_HPO_4_ (1 mM, pH 7.0), KCl (5 mM). For each oligonucleotide sample, a UV spectrum was recorded above and below its melting temperature (T_m_). All experiments were performed on a Jasco V 750 UV/Vis spectrophotometer (Jasco, Tokyo, Japan) using quartz cuvettes with an optical path of 1 cm and at 25 μM strand concentration. Absorbance spectra were recorded in the 220–340 nm range, with a scan speed of 200 nm min^−1^ and a data interval of 1 nm. The difference between the UV spectra at high (95 °C) and low (20 °C) temperatures was defined as the TDS; this represents the spectral difference between the unfolded and folded forms. The temperature (20 or 95 °C) was kept constant with a thermostatic circulating water bath for cell holders (Jasco CTU-100). The thermal difference spectra were normalized (+1 for the highest positive peak).

### 4.5. Gel Electrophoresis

All oligonucleotides were analyzed by non-denaturing PAGE. All oligonucleotide samples were prepared at an ODN concentration of 1 mM using a potassium phosphate buffer (10 mM KH_2_PO_4_/K_2_HPO_4_, 70 mM KCl, pH 7.0) and submitted to the annealing procedure (heating at 90 °C and slowly cooling at room temperature). Each oligonucleotide was loaded on a 20% polyacrylamide gel containing Tris–Borate-EDTA (TBE) 2.5× and KCl 20 mM. The run buffer was TBE 1× containing 50 mM KCl. For all samples, a solution of glycerol/TBE 10× was added just before loading. Electrophoresis was performed at 8 V/cm at a temperature close to 10 °C. Bands were visualized by UV shadowing.

### 4.6. Nuclease Stability Assay

Nuclease stability assay of all ODNs was conducted in 10% Fetal Bovine Serum (FBS) diluted with Dulbecco’s Modified Eagle’s Medium (DMEM) at 37 °C and studied by CD analysis. Approximately 7 nmol of stock solution of each ODN (~1 O.D.U.) was evaporated to dryness under reduced pressure and then incubated with 250 μL 10% FBS at 37 °C. The degradation patterns were analyzed by monitoring the CD signal decrease in each sample at 37 °C, as a function of time. CD spectra at 0, 6, 24, 48 and 72 h for all ODNs were recorded at 37 °C using a Jasco 715 spectrophotometer equipped with a Peltier temperature control system (Jasco, Tokyo, Japan). Data were collected from 240 to 320 nm with a 1 s response time and a 1 nm bandwidth using a 0.1 cm quartz cuvette. Each spectrum shown is corrected for the spectrum of the reaction medium (10% FBS in DMEM).

### 4.7. Cell Cultures

Human breast cancer cell line MDA-MB-231 (cat. no. HTB-26) and human prostate cancer cell line DU145 (cat. no. HTB-81) were purchased from the American Type Culture Collection (ATCC, Manassas, VA, USA). Cells were cultured in DMEM (Sigma-Aldrich, Milan, Italy; cat. no. D6546) supplemented with 10% fetal bovine serum (FBS) (Gibco, Milan, Italy; cat. no. A4736301), penicillin (100 U/mL) and streptomycin (100 μg/mL) (cat. no. 30-002-CI), 2 mmol/L L-glutamine (cat. no. 25-005-CI) and 0.01 M HEPES buffer (cat. no. 25-060-CI) (all from Corning, Manassas, VA, USA) and placed at 37 °C in a humidified incubator containing 5% CO_2_.

### 4.8. MTT Assay

Cell proliferation was measured by MTT (3-(4,5-dimethylthiazol-2-yl)-2,5-diphenyltetrazolium bromide) assay. MDA-MB-231 and DU145 cells were seeded on 96-well plates (6 × 10^3^ cells/well). After 24 h, cells were treated with **STAT**, **STAT T1**, **STAT T2**, **STAT T3**, **STAT T4**, **STATB**, **STATB T1**, **STATB T2**, **STATB T3**, **STATB T4** and **STATB T5** (10 and 30 µM) for 48 and 72 h before adding MTT (cat. M5655, Merk, Italy) (final concentration 5 mg/mL in PBS). Cells were incubated for an additional 3 h at 37 °C. After this time, cells were lysed, and formazan salts resulting from MTT reduction were solubilized with a solution containing 50% (*v*/*v*) N,N-dimethyl formamide, 20% (*w*/*v*) sodium dodecylsulfate with an adjusted pH of 4.5. The absorbance was measured using a microplate spectrophotometer (Thermo Scientific Multiskan GO, Thermo Fisher Scientific, Waltham, MA, USA) equipped with a 570-nm filter.

### 4.9. RNA Purification and Quantitative Real-Time PCR

MDA-MB-231 and DU145 cells were seeded (106 cells/well) in 100 mm culture dishes and, after 24 h, treated or not with **STAT** and **STATB** (30 µM) for 48 and 72 h. Total RNA was isolated from cells using the QIAzol Lysis Reagent according to the manufacturer’s instructions (Cat. 79306, Qiagen, Hilden, Germany). The purity and quantity of each purified RNA were evaluated considering the ratio between readings at 260/280 nm using an Eppendorf BioPhotometer and Nanodrop apparatus (Thermo Fisher Scientific, MA, USA). Purified mRNA was reverse-transcribed using iScript Reverse Transcription Supermix for RT-qPCR (cat. 1708841, Bio-Rad, Segrate (MI), Italy). qPCR was carried out in a CFX96 real-time PCR detection system (Bio-Rad) with the use of specific primers (Cyclin D1 5′-GCTGCGAAGTGGAAACCATC-3′, 5′-CCTTCTGCACACATTTGAA-3′; BAX 5′-CCCGAGAGGTCTTTTTCCGAG-3′, 5′-CCAGCCCATGATGGTTCTGAT-3′; c-Myc 5′-GGCTCCTGGCAAAAGGTCA-3′, 5′-CTGCGTAGTTGTGCTGATGT-3′; CDC25A 5′-CTCCTCCGAGTCAACAGATTCA-3′, 5′-CAACAGCTTCTGAGGTAGGGA-3′; IL-1β 5′-ATGATGGCTTATTACAGTGGCAA-3′, 5′-GTCGGAGATTCGTAGCTGGA-3′; IL-6 5′-AGACAGCCACTCACCTCTTCAG-3′, 5′-TTCTGCCAGTGCCTCTTTGCTG-3′; COX-2 5′-TAAGTGCGATTGTACCCGGAC-3′, 5′-TTTGTAGCCATAGTCAGCATTGT-3′; VEGF 5′-AGGGCAGAATCATCACGAAGT-3′, 5′-AGGGTCTCGATTGGATGGCA-3′; αSMA 5′-CTATGCCTCTGGACGCACAACT-3′, 5′-CAGATCCAGACGCATGATGGCA-3′) and SYBR Green master mix kit (cat. 1725271, Bio-Rad). Real-Time PCR cycling protocol was: polymerase activation and DNA denaturation 95 °C for 30 s, amplification 40 cycles, denaturation 95 °C for 15 s, annealing and extension 60 °C for 30 s, plate read at 60 °C, melt-curve analysis 65–95 °C 0.5 °C increment, 5 s/step. The housekeeping gene (ribosomal protein S16) was used as an internal control to normalize the CT values using the 2^−ΔΔCt^ formula.

### 4.10. Statistical Analysis

Data were expressed as mean ± SEM of *n* = 3 experiments. Data were analyzed and presented using GraphPad Prism 8.2.0 software (San Diego, CA, USA). Significance was determined using Student’s two-tailed *t*-test. Results were considered significant at *p* values less than 0.05 and are labelled with a single asterisk. In addition, *p*-values lower than 0.01 and 0.001 are indicated with double and triple asterisks, respectively.

## 5. Conclusions

In summary, we investigated the structural and biological features of two series of G4 forming oligonucleotides, **STAT** and **STATB** sequences, and their respective analogues, which contain a single site-specific replacement of the C loop residues with a T one. This study aims to identify new aptamers modulating their biological properties, particularly their ability to affect the IL6/STAT3 biochemical pathway, without affecting their stability. The evaluation of the structural features indicated that all derivatives preserve the ability to fold in very stable G4 structures that are strictly similar to that of the unmodified aptamer T40214. Furthermore, the antiproliferative activities against MDA-MB-231 and DU145 cancer cell lines, and biological degradability of **STAT** and **STATB** series were analyzed. The most interesting antiproliferative effects were registered at 72 h at 30 µM; under these conditions, all investigated ODNs showed a similar biological activity on both cell lines, revealing a marked antiproliferative power. The results concerning the nuclease stability indicated that, after 72 h in fetal bovine serum, **STAT** series derivatives mostly retain their structures, while the **STATB** series analogues show a lower percentage of undegraded species. Anyway, their nuclease resistance, to some extent, suggests that the structured species for all ODNs, which are probably most responsible for the antiproliferative activity, persist over time. Finally, a transcriptomic analysis aimed to evaluate **STAT** and **STATB**’s influence on the expression of a large plethora of genes in MDA-MB-231 cells, suggested the potential involvement of both aptamers in the modulation of IL-6/STAT3 pathway, leading to their interfering in different biological processes. These data provide new tools that could affect an interesting biochemical pathway and help to develop novel anticancer and anti-inflammatory drugs.

## Figures and Tables

**Table 1:** Sequences and melting temperatures (T_m_) at low potassium concentration of the investigated ODNs.

Oligonucleotide	Sequence	T_m_ (°C) ± 1
**STAT**	5′-GGGCGGGCGGGCGGGC-3′	82
**STAT T1**	5′-GGG**T**GGGCGGGCGGGC-3′	87
**STAT T2**	5′-GGGCGGG**T**GGGCGGGC-3′	82
**STAT T3**	5′-GGGCGGGCGGG**T**GGGC-3′	85
**STAT T4**	5′-GGGCGGGCGGGCGGG**T**-3′	87
**STATB**	5′-GCGGCGGGCGGGCGGGC-3′	63
**STATB T1**	5′-G**T**GGCGGGCGGGCGGGC-3′	67
**STATB T2**	5′-GCGG**T**GGGCGGGCGGGC-3′	67
**STATB T3**	5′-GCGGCGGG**T**GGGCGGGC-3′	67
**STATB T4**	5′-GCGGCGGGCGGG**T**GGGC-3′	74
**STATB T5**	5′-GCGGCGGGCGGGCGGG**T**-3′	72

## Data Availability

The data presented in this study are available on request from the corresponding author.

## Supplementary Materials

The supporting information can be downloaded at: https://www.mdpi.com/article/10.3390/ijms24119524/s1.
