# Supplementary material for: Structural and Biological Features of G-Quadruplex Aptamers as Promising Inhibitors of the STAT3 Signaling Pathway
Source: Int J Mol Sci. 2023 May 30;24(11):9524. doi: 10.3390/ijms24119524 (PMC10253491; doi:10.3390/ijms24119524)
Supplement: Supplementary file 1 [file ijms-24-09524-s001.zip › ijms-2417716-supplementary.pdf]

Supplementary Material

Structural and biological features of G-quadruplex aptamers as promising inhibitors of the STAT3 signaling pathway.

Veronica Esposito, Daniela Benigno, Ivana Bello, Elisabetta Panza, Mariarosaria Bucci, Antonella Virgilio* and Aldo Galeone

Department of Pharmacy, University of Naples Federico II, Via D. Montesano 49, I-80131 Naples, Italy

* Corresponding Author: (AV), e-mail: antonella.virgilio@unina.it; tel.: +39081678536

Table of contents

CD melting profiles.

PAGE.

CD spectra in 10% Fetal Bovine Serum (FBS).

MTT assay in DU145 cells.

Tables of undegraded species percentages at 72h.

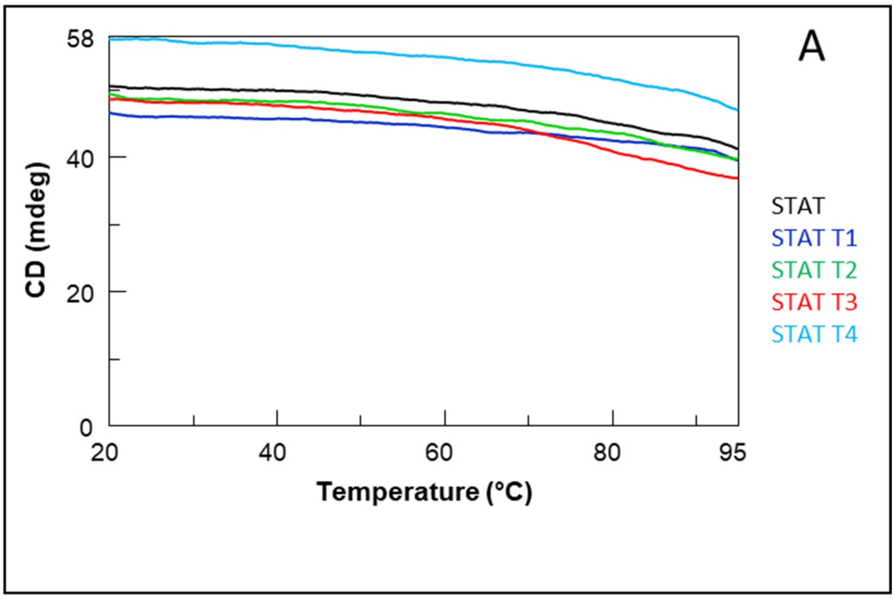

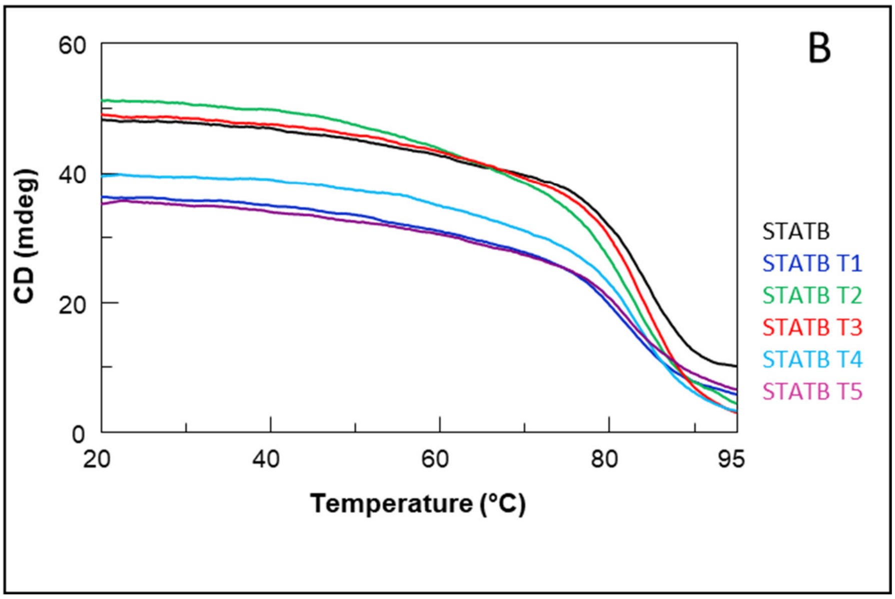

Figure S1. CD melting profiles of **STAT** (panel A) and **STATB** (panel B) series registered as a function of temperature for all modified quadruplexes at their maximum Cotton effect wavelengths. CD data were recorded in a 0.1 cm pathlength cuvette with a scan rate of 30°C/h at 50 μM ODN strand concentration in potassium phosphate buffer (10 mM KH_2PO_4/K_2HPO_4, 70 mM KCl, pH 7.0).

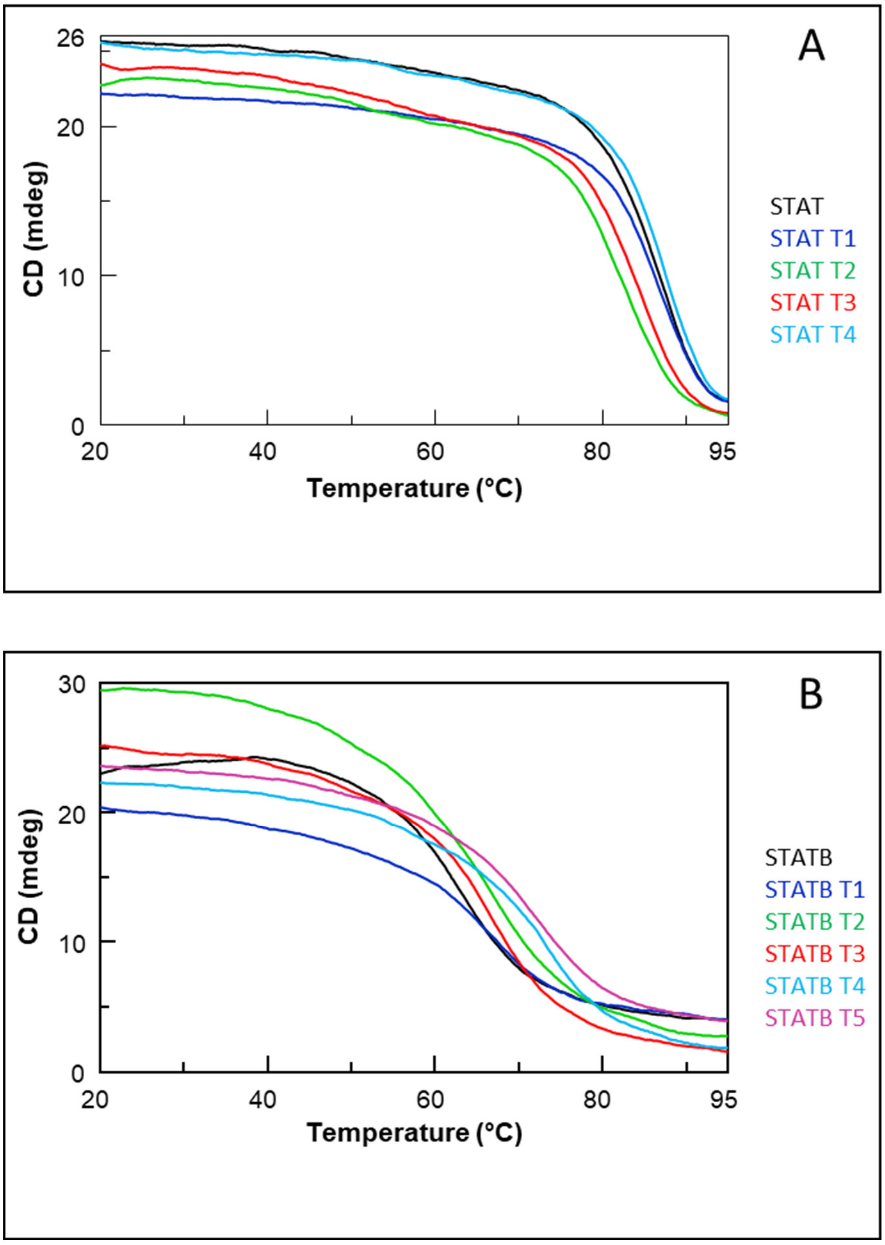

Figure S2. CD melting profiles of **STAT** (panel A) and **STATB** (panel B) series registered as a function of temperature for all modified quadruplexes at their maximum Cotton effect wavelengths. CD data were recorded in a 0.1 cm pathlength cuvette with a scan rate of 30°C/h at 50 μM ODN strand concentration in potassium phosphate buffer (1 mM KH_2PO_4/K_2HPO_4, 5 mM KCl, pH 7.0).

A

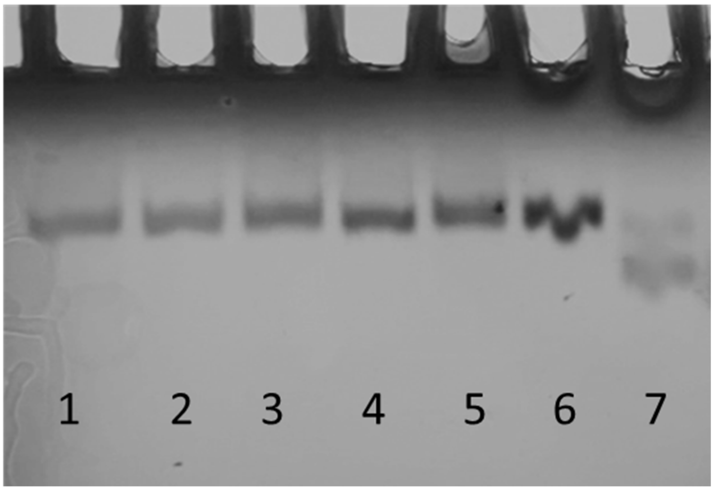

B

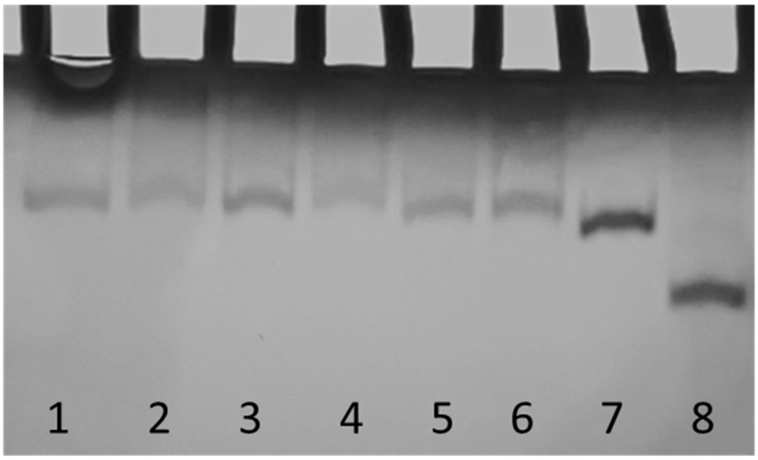

Figure S3. PAGE analysis of **STAT** and its investigated analogues (panel A). Lane 1: **STAT**; lane 2: STAT T1; lane 3: STAT T2; lane 4: STAT T3; lane 5: STAT T4; lane 6: INT; lane 7: TT-INT. PAGE analysis of **STATB** and its investigated analogues (panel B). Lane 1: **STATB**; lane 2: STATB T1; lane 3: STATB T2; lane 4: STATB T3; lane 5: STATB T4; lane 6: STATB T5; lane 7: INTB; lane 8: TT-INTB. INT, TT-INT, INTB and TT-INTB have been used as references. See Materials and Methods for experimental details.

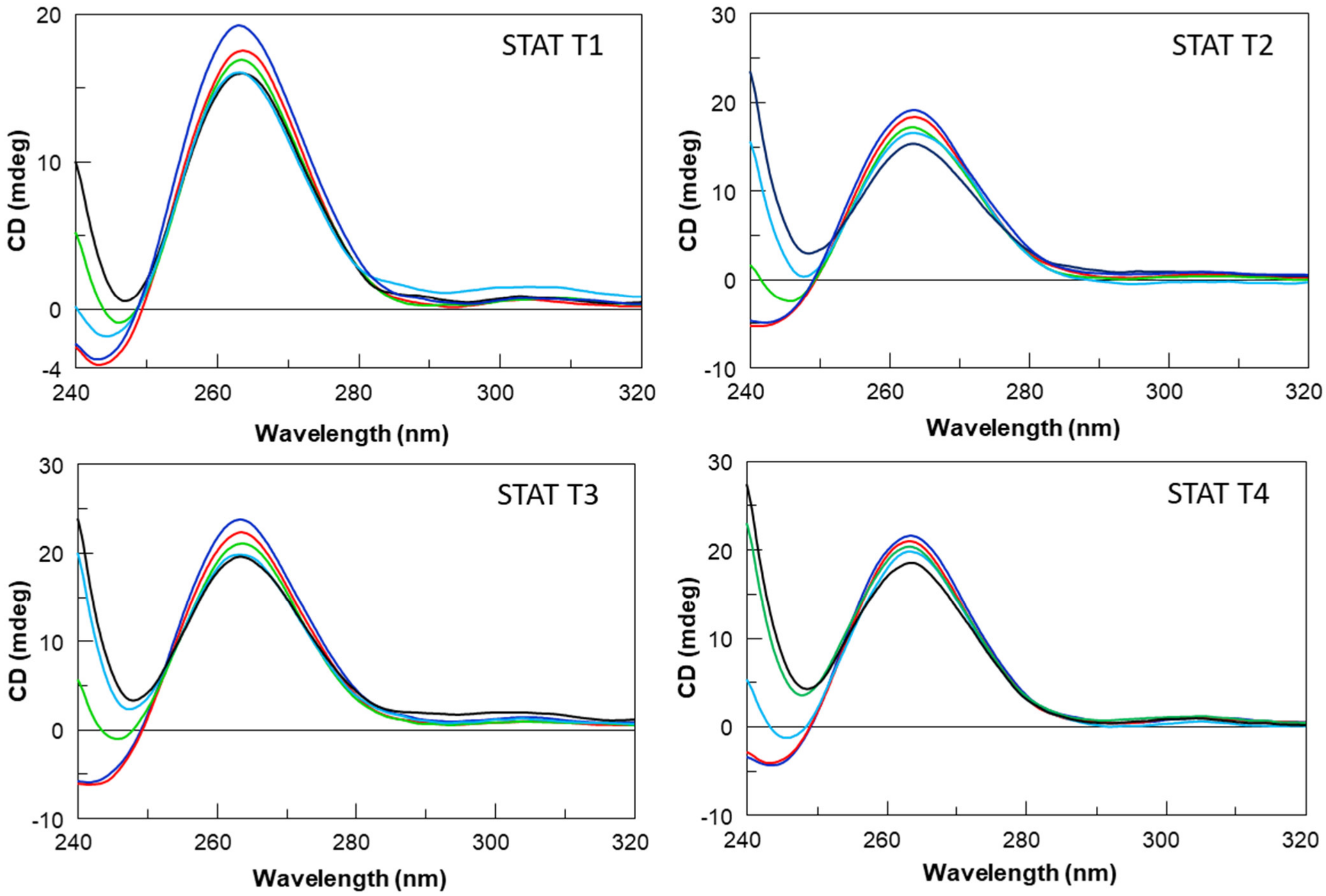

Figure S4. CD spectra at 37°C of **STAT** analogues in 10% Fetal Bovine Serum (FBS) diluted with Dulbecco's Modified Eagle's Medium (DMEM), registered at different time: 0 h (blue), 6 h (red), 24 h (green), 48 h (light blue), 72 h (black). See the main text and the Materials and Methods section for details.

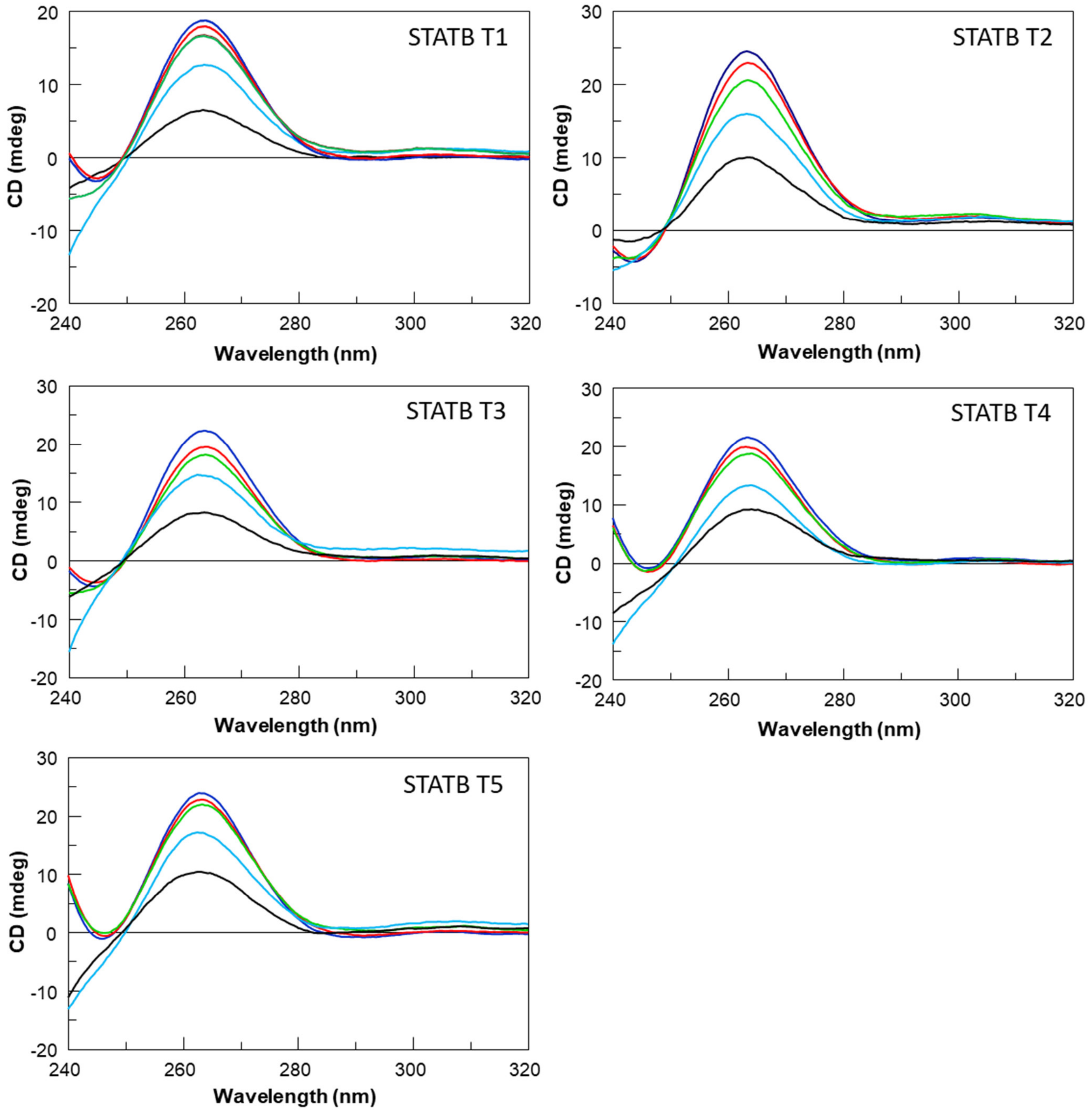

Figure S5. CD spectra at 37°C of **STATB** analogues in 10% Fetal Bovine Serum (FBS) diluted with Dulbecco's Modified Eagle's Medium (DMEM), registered at different time: 0 h (blue), 6 h (red), 24 h (green), 48 h (light blue), 72 h (black). See the main text and the Materials and Methods section for details.

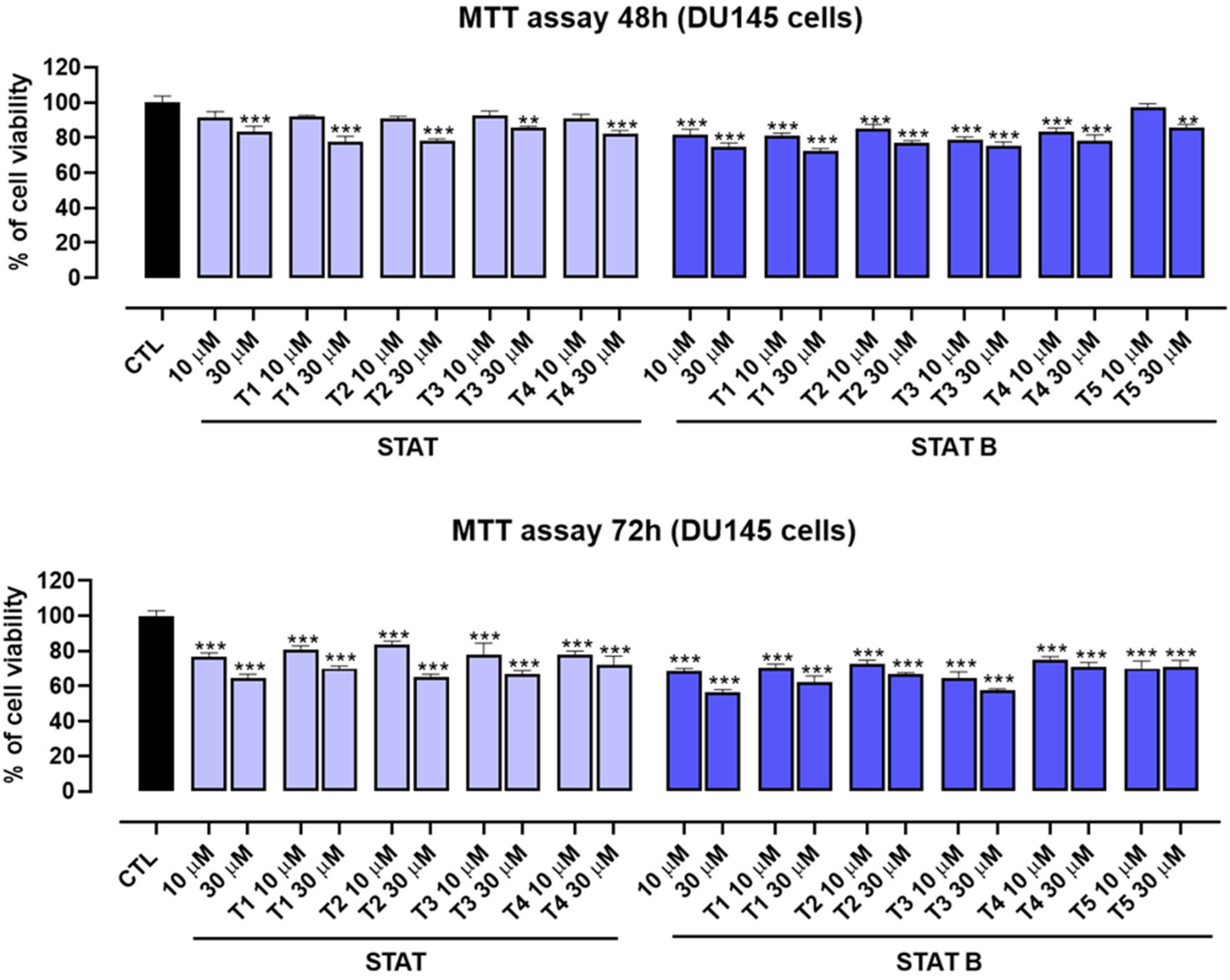

Figure S6. Effects of **STAT** and **STATB** series on DU145 cell proliferation. Cell proliferation was measured using the MTT assay and evaluated at 48 and 72 h. Each experiment (*n* = 3) was run in quadruplicate. **P < 0.01; ***P < 0.001 vs. CTL.

Sample	Undegraded specie at 72h (%)
STAT	84
STAT T1	83
STAT T2	80
STAT T3	82
STAT T4	85

Table S1. Percentages of undegraded folded species (G-quadruplexes) persistent at 37°C in each sample solution (10% FBS in DMEM) at 72h (**STAT** series).

Sample	Undegraded specie at 72h (%)
STATB	28
STATB T1	35
STATB T2	41
STATB T3	37
STATB T4	42
STATB T5	44

Table S2. Percentages of undegraded folded species (G-quadruplexes) persistent at 37°C in each sample solution (10% FBS in DMEM) at 72h (**STATB** series).
